# Does External Innovation Promote the Exports of Private Enterprises? A Market Stakeholder Perspective

**DOI:** 10.3389/fpsyg.2022.913026

**Published:** 2022-05-27

**Authors:** Siyu Chen, Xiaojing Jiang, Yujia Wan, Jie Hao

**Affiliations:** ^1^School of Business Administration, Zhejiang Gongshang University, Hangzhou, China; ^2^School of Economics, Nankai University, Tianjin, China; ^3^School of Accounting, Zhejiang Gongshang University, Hangzhou, China

**Keywords:** stakeholder, external innovation, private enterprises, export propensity, export intensity, export scale

## Abstract

Using the theoretical perspective of market stakeholders, we analyze the impact of external innovations from upstream enterprises, downstream enterprises, and competitors on the exports of private enterprises. By using data from the China Industrial Enterprises Database, we find that external innovations from upstream suppliers, downstream customers and horizontal competitors show positive impacts on the export propensity, intensity and scale for private enterprises. The results of a heterogeneity analysis indicate that the positive relationships between the external innovations of stakeholders and the exports of private enterprises are stable in different factor-intensive industries. In addition, while the exports of private enterprises are positively correlated with their external innovations in the eastern and central regions, this relationship is not significant in the western region. Further, the mechanism analysis confirms that enterprise innovation played an important mediating role for the external innovations of stakeholders to promote the exports of private enterprises. This study provides useful policy implications for enhancing the export competitiveness of private enterprises.

## Introduction

In recent years, the prices of the exports of Chinese enterprises have been gradually becoming less competitive due to the continuous increases in the costs of land, wages, and other factors. While exports of traditional manufacturing and services such as clothing are facing fierce competition from countries and regions such as Southeast Asia, India, South America and Africa, China’s export enterprises face increasingly stringent trade blockades and technology restrictions on high-tech intermediates (Essaji, 2008; Bao and Chen, 2013). Private enterprises have become the backbone of exports as their contribution to overall import and export growth has exceeded 50%. Thus, how to maintain and expand the exports of private enterprises has become a hot topic for both policymakers and scholars.

However, intensifying trade wars and tech blockades have put enterprises that lack independent innovation at greater risk of trade disruptions. Therefore, improving the competitiveness of enterprises’ exported products and services through innovation has become a key path for private enterprises to move toward a higher position in the global value chain. According to the endogenous growth theory, innovation is the key factor that determines the export competitiveness of enterprises (Grossman and Helpman, 1993). Innovation helps to meet the diversified needs of overseas consumers, thus enhancing the price competitiveness of exported products (Dai et al., 2020). It also helps to break the technology blockade and patent protection in developed countries, and it enables enterprises to export to a higher value chain (Jacobides et al., 2006). Overall, upgrading technology and product diversity through independent innovation can reduce production costs and overcome a foreign technology blockade, which further helps private enterprises obtain export advantages (Caldera, 2010). This view has been supported by extant studies, which have found that enterprise innovation has been the main driving force to promote upgrading the quality of enterprise exports and realizing the steady growth of export volume (Cockburn et al., 2016).

Prior studies have mainly focused on how an enterprise’s own innovation has affected its exports, and they rarely have examined the role of the external innovation of market stakeholders. The term “market stakeholders” has been used to refer to upstream suppliers, horizontal competitors and downstream customers, which can affect other enterprises or be affected by others through market exchange (Sharma and Henriques, 2005; von der Heidt and Scott, 2011; Li et al., 2018).

In this study, we propose that the exports of private enterprise are not only affected by their own innovations, but also closely related to the external innovations of their market stakeholders. First, overseas customers’ preferences and demands for the exported products are closely related to the innovation of the products, which depends not only on the innovation efforts of the enterprises themselves, but also on the innovation of the upstream and downstream enterprises and even the competitive enterprises.

For instance, the technological breakthroughs of the domestic upstream enterprises in the cutting-edge equipment, basic components and special materials can increase the technological complexity of enterprises’ exported products and decrease the cost of intermediate products by breaking the foreign technological monopoly, thus enhancing the competitive advantages of export enterprises (Spencer and Raubitschek, 1996; Edeh et al., 2020). Moreover, the external innovations of market stakeholders may promote the exports of private enterprises by promoting the latter’s innovation: the external innovations of market stakeholders are thought to promote the innovation of enterprises through mechanisms such as resource exchange, knowledge spillovers and pressure transmission (Li et al., 2018). And the innovation of enterprises can promote their exports (Faruq, 2010). Therefore, enterprise innovation may be an important mediating mechanism for external innovation to promote the exports of private enterprises.

For our analysis, we used data from the China Industrial Enterprise Database and calculated the degrees of external innovation of upstream suppliers, downstream customers and competitive enterprises (Li et al., 2018). We then undertook a three-phrased approach. First, we examined the effects of three types of external innovation on the export propensity, volume and intensity of private enterprises. Second, we analyzed the heterogeneity of the relationship between external innovations and private enterprise exports based on industrial and regional factors, which enriched the conclusions of our study. Third, we examined the mediating roles of enterprise innovation in the relationships between three types of external innovation and the exports of private enterprises. These conclusions provide useful policy implications for the comprehensive impacts of external innovations on the exports from the perspective of the value chain.

Our paper offers several contributions to the literature. First, by investigating the external innovation of stakeholders, we help expand our understanding of how stakeholder theory impacts enterprise behavior. According to the classical stakeholder theory, the competitive advantage of enterprises depends not only on their internal resources and capabilities, but also on the resource supply capacity and the quality of suppliers, customers, creditors and other stakeholders. On this basis, we directly locate the role of stakeholders in the field of innovation, and we discuss the relationship between external innovation and enterprise exports. While the extant literature has focused on whether the stakeholder orientation of enterprises contributes to their innovation (Gould, 2012; Flammer and Kacperczyk, 2016), we aimed to investigate how a stakeholder’s innovation affects enterprise behavior (i.e., exports). Although Li et al. (2018) explored the relationship between the external innovation of market stakeholders and enterprise innovation, it seems that very little research has been done to link the external innovation of stakeholders with the exports of private enterprises. In this paper, we propose that stakeholders’ external innovations can enhance the core competitiveness of private enterprises and thus promote their exports. This view enriches our understanding of the effect of stakeholders’ theory on the behavior of enterprises.

Second, prior research has noted that technological innovation is an important method of enhancing the exports of private enterprises. We propose that the enhancement of export competitiveness of private enterprises is not only based on their own innovation, but also closely related to the external innovation of their market stakeholders. In other words, the innovation activities of upstream suppliers, downstream customers and competitors can improve the export performance of private enterprises. These conclusions supplement the prior literature, which has largely ignored the influence of stakeholders on enterprise exports. Thus, we provide a beneficial inspiration for private enterprises to promote their exports by encouraging external innovation of stakeholders.

The remainder of this paper is arranged as follows: The second section describes the literature and hypotheses; the third section discusses the research model and variable descriptions; the fourth section reports results; and the fifth section provides our conclusions and implications.

## Literature Review and Hypotheses

Since China joined the World Trade Organization in December 2001, Chinese enterprises – especially private enterprises – have quickly entered the global market with the advantages of low costs and a flexible response to market demands. Chinese private enterprises have mainly engaged in low-end value chain activities with low-tech and labor-intensive characteristics (Levchenko, 2007; Nunn, 2007; Zhang et al., 2021), and their independent technological capabilities have been relatively weak. Thus, Hanson et al. (2005) suggested that the comparative advantage of Chinese enterprises in exports depends on the relatively low cost of labor and other factors, rather than the ability of independent innovation.

However, due to the continuous increase in factor costs (e.g., wages), the high growth of labor-intensive product exports is difficult for Chinese enterprises to maintain (Faruq, 2010; Zhang et al., 2021). Hence, the role of independent innovation in enterprise exports is increasingly valued by scholars in the field. Some studies have suggested that if an enterprise lacks independent innovation and core technology and relies too much on foreign technology transfer, it may be locked in the dilemma of a low-end value chain and mainly export primary processed products (Spencer and Raubitschek, 1996). Many studies based on the endogenous growth theory have proposed that independent innovation can help enterprises obtain export advantages (Caldera, 2010), as technological innovation can enhance the competitive advantage of products (Jin and Cho, 2018). The innovative activities of enterprises promote the upgrading of product appearances and functions, which helps enterprises enhance competitive advantages (Liu and Xie, 2020; Zhang et al., 2021). In particular, some primary innovations may help enterprises create a “blue ocean market” and thus break through the “low-end locking” trade dilemma (Aghion et al., 2005). However, enterprises can improve production efficiency and reduce production costs by transforming the production process in the hope of successfully competing on export prices (Yeaple, 2005). Therefore, enterprise innovation has been considered as a key factor in promoting the exports of private enterprises.

Based on the extant research, our paper investigates how the exports of private enterprises are affected by external innovation of market stakeholders. We propose that the external innovations of stakeholders can directly affect the exports of private enterprises. First, the innovation of upstream suppliers can provide enterprises with higher-quality raw materials and components, which is expected to improve the diversity and quality of exported products. In particular, breakthroughs in upstream core technologies can often disrupt foreign monopolies and significantly lower the prices of intermediate products imported from abroad, thus reducing the production costs of exporting enterprises (Spencer and Raubitschek, 1996). Second, the innovation of competitors and downstream enterprises can help to enhance the overall image and reputation of local enterprises, which may form a reputation spillover effect and promote the exports of enterprises. Therefore, we propose:

H1: *External innovations by market stakeholders can significantly promote the exports of private enterprises.*

The stakeholder theory posits that external innovation of market stakeholders can promote enterprise innovation (Li et al., 2018) – that is, the external innovation of suppliers, customers and competitors promotes enterprise innovation through resource exchange, knowledge spillover and pressure transmission.

In terms of resource exchange and knowledge spillover mechanisms, upstream suppliers have the motivation to provide and share their innovative achievements to the enterprises, hoping to improve the latter’s product competitiveness and increase their sales and establish a more stable supply-demand chain relationship (Takeishi, 2001). Similarly, to promote enterprises to provide higher quality products, local downstream customers are motivated to share their innovative ideas and achievements in product development, quality control and process design with enterprises (Li et al., 2018). An enterprise can also benefit by imitating and tracking competitors’ innovations (Mowery et al., 1996).

In terms of a pressure transmission mechanism, the external innovation of stakeholders will bring innovation pressure to the enterprise. For example, the innovation of competitors will bring greater competitive pressure to the enterprise, while the innovation of upstream and downstream enterprises may also drive the enterprise to update its own technology and process; otherwise, customers and suppliers may switch to cooperate with other enterprises (Li et al., 2018). Thus, external innovation may force the enterprises to strengthen innovation activities.

Further, external innovation may promote enterprises’ innovation by activating social norms of managers. Specifically, social norms describe that an individual’s decision is often influenced by what most people actually do or ought to do (Cialdini et al., 1990; Yin et al., 2021). Thus, if market stakeholders such as competitors engage in extensive innovation activities, the enterprise managers may regard innovation activities as one types of social norm, and thus enhance the innovation activities of their own enterprises driven by the force of norm compliance.

Moreover, the extant literature has proposed that independent innovation of enterprises helps to promote their exports (Yeaple, 2005). Therefore, we propose that an enterprise’s innovation may play an important mediating role between the external innovations of market stakeholders and exports of private enterprises – that is, external innovation can promote an enterprise’s exports by promoting the latter’s innovation.

H2: *The enterprise innovation plays a significant mediating role in the relationship between the external innovations by market stakeholders and the exports of private enterprises.*

**FIGURE 1:**
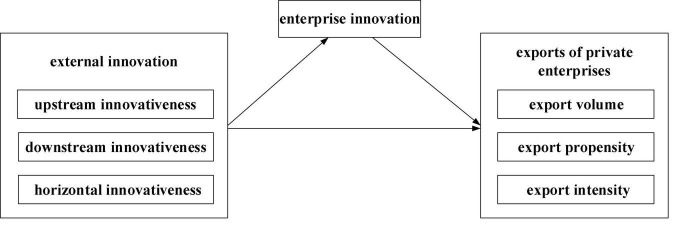
Conceptual model.

Accordingly, Figure 1 shows the conceptual model of this study.

## Research Design

### Data Description

We used data from the 1998–2010 China Industrial Enterprise Database released by the National Bureau of Statistics. However, data from 2004 and 2008 were dropped because of missing information for new product output (the independent variable). The enterprise sample covered 31 provinces and 43 industrial industries. Following Cai and Liu (2009), we deleted the samples with missing assets as well as those failing to meet the accounting standards, such as the samples in which total assets were less than current assets or net fixed assets, and the samples in which total assets were negative.

### Variables

#### Dependent Variables

We measured the export behavior of private enterprises by three variables: *export volume*, *export propensity*, and *export intensity*. Export volume was measured by export delivery value. Export propensity was a dummy variable, which equaled 1 when the export delivery value was greater than 0; otherwise, it equaled 0. To control the impact of scale factors, we selected export intensity as a dependent variable. Export intensity was the ratio of the enterprise’s export delivery value to the sales value; the higher the export intensity, the more inclined the enterprise would be to export.

#### Independent Variables

Similar to Li et al. (2018), we calculated three independent variables, i.e., *upstream innovativeness*, *downstream innovativeness* and *horizontal innovativeness*, to measure the degrees of external innovation of upstream enterprises (represented by suppliers), downstream enterprises (represented by customers) and competitive enterprises (in the same industry), respectively. The above independent variables were measured at the region-industry level – that is, the market stakeholders were from the same region and related industries (upstream industry, downstream industry and the same industry) the target export enterprise. The industry codes in this paper were based on the GB/T4754-2002.

Specifically, *horizontal innovativeness* was used to measure the degree of innovation of competitors, which was measured by the sum of new product output values of all enterprises in the same region-industry except for the target enterprises. The *upstream innovativeness* was the weighted average of the new product output value of all upstream industries in the same area, as shown in formula (1). *Upstream New Product Output_i_* represented the new product output value of upstream industry *i* in the region, and *a*_*i*_ was the ratio of the intermediates from the upstream industry *i* to the total intermediates. Compared to simply calculating the sum of new product output values of all upstream industries, the weighted average method of (1) can better describe the impact of upstream industry innovation on the industry.

Similarly, the calculation method of downstream innovativeness is shown in formula (2), where β_*i*_ represented the ratio of intermediate output (provided by the industry in which the target enterprise was located in relation to the downstream industry *i*) to total intermediate output (provided by the industry in which the target enterprise was located in relation to all downstream industries). The intermediate input of the upstream industry to the industry and the intermediate output provided by the industry to the downstream industry were from the national input-output basic table (the basic flow table in the input-output table) compiled by the National Bureau of Statistics in 2002, 2007, and 2012.


(1) Upstream Innovativeness=Σ aUi*pstream New Product Outputi

(2) Downstream Innovativeness=Σ βDi*ownstream New Product Outputi

#### Control Variables

We used following control variables. (1) *Market concentration ratio* was calculated using the Herfindahl-Hirschman index (HHI) of a province. The lower the market concentration, the stronger the competition between enterprises in the same industry in the region, which may promote enterprises to seek overseas markets. (2) *Enterprise scale* was also used because it affects the production efficiency and anti-risk capacities of an enterprise (Ilmakunnas and Nurmi, 2010; Dai et al., 2020). A larger firm may have a higher export propensity and scale. In accordance with the “Measures for the Classification of Large, Medium and Small-sized Enterprises in Statistics (temporary)” issued by the National Bureau of Statistics, if an enterprise has more than 2,000 employees, the sales were more than 300 million yuan and the total assets were more than 400 million yuan, then the enterprise scale was 3; if the number of employees were between 300 and 2,000, the sales were between 30 million and 300 million yuan, and the total assets were between 40 million and 400 million yuan, then the enterprise scale value was 2; if the number of employees were less than 300, and the sales and total assets were less than 30 million and 40 million respectively, the enterprise scale value was 0; in all other cases, the enterprise scale value was 1. (3) *Enterprise age*. The longer the enterprise had been in existence, the greater the possibility of exports (Disney et al., 2003). Therefore, the time distance from the year of enterprise establishment to the present was taken as the control variable. (4) We took *corporate financing constraints* and *capital intensity* as two control variables (Bellone et al., 2010; Zhang et al., 2018). We used the ratio of corporate accounts receivable to sales revenue to measure financing constraints, and we used the ratio of the annual average net value of fixed assets to the number of employees to measure corporate capital intensity. The two control variables were logarithmically processed. In addition, to weaken the influence of outliers on the regression results, we winsorized at the 1 and 99% levels for the above variables.

## Results

### Main Effect

The basic estimation model was:


$$Export\;Behaviors_{pif}=a_{0}+\beta Innovativeness_{pi}+\beta^{\prime }X+\mu_{year}+\mu_{firm}+\varepsilon_{pif}$$

In this model, *p, i*, and *f* represented different provinces, industries and enterprises, *a*_0_ was the intercept term; *u*_*year*_ and *u*_*firm*_ represented the year fixed effect (Year FE), firm fixed effect (Firm FE), *ε_*pif*_* was the random disturbance term, and X represented the six control variables. *Export Behaviors_*pif*_* represented three enterprise-level dependent variables for measuring enterprise exports, namely export propensity, volume and intensity. *Innovativeness*_*pi*_ included three independent variables: upstream, downstream and horizontal innovation. Three dependent variables and three independent variables were combined to obtain nine regression estimation models, as shown in Table 1. In Table 1, models (1) – (3), (4) – (6), (7) – (9) display the regression results of external innovation on export propensity, volume and intensity, respectively.

**TABLE 1:** Regression results of upstream, downstream, and horizontal innovation on exports of private enterprises.

Model	(1)	(2)	(3)	(4)	(5)	(6)	(7)	(8)	(9)
			
Variables	Export propensity			Export volume			Export intensity		
Upstream innovativeness	0.7180*** (0.012)			0.4647*** (0.007)			0.0059*** (0.000)		
Downstream innovativeness		0.6459*** (0.012)			0.4092*** (0.007)			0.0047*** (0.000)	
Horizontal innovativeness			0.4130*** (0.009)			0.1033*** (0.002)			0.0012*** (0.000)
Market concentration	0.7861** (0.396)	1.0253*** (0.393)	2.1589*** (0.399)	0.3341*** (0.108)	0.3872*** (0.108)	0.4336*** (0.109)	–0.0016 (0.005)	–0.0011 (0.005)	–0.0006 (0.005)
Enterprise size	0.6597*** (0.092)	0.6647*** (0.091)	0.6635*** (0.091)	0.4615** (0.052)	0.4682*** (0.052)	0.4667*** (0.052)	0.0113*** (0.002)	0.0114*** (0.002)	0.0113*** (0.002)
Enterprise age	0.0120*** (0.003)	0.0098*** (0.003)	0.0101*** (0.003)	0.0036*** (0.001)	0.0031** (0.001)	0.0037*** (0.001)	0.0001** (0.000)	0.0001** (0.000)	0.0001** (0.000)
Enterprise finance constraints	–0.0176** (0.009)	–0.0165* (0.009)	–0.0214** (0.009)	–0.0266*** (0.003)	–0.0261*** (0.003)	–0.0250*** (0.003)	–0.0004** (0.000)	–0.0004** (0.000)	–0.0003* (0.000)
Enterprise capital intensity	–0.0468*** (0.011)	–0.0363*** (0.011)	–0.0453*** (0.011)	–0.0279*** (0.005)	–0.0251*** (0.005)	–0.0302*** (0.005)	–0.0017*** (0.000)	–0.0017*** (0.000)	–0.0018*** (0.000)
Enterprise fixed effect	Yes	Yes	Yes	Yes	Yes	Yes	Yes	Yes	Yes
Year fixed effect	Yes	Yes	Yes	Yes	Yes	Yes	Yes	Yes	Yes
*N*	102319	102319	102319	701173	701173	701173	700679	700679	700679
*R* ^2^				0.8332	0.8326	0.8310	0.8586	0.8585	0.8584

Robust standard errors are reported in parentheses. ***p < 0.01; **p < 0.05; *p < 0.1. Logit models are used in (1) – (3).

The results in Table 1 demonstrate that the external innovations of stakeholders were helpful to promote the exports of private enterprises. Whether it were upstream suppliers, downstream customers or horizontal competitors, their innovation activities could promote the export tendency, volume and intensity of private enterprises, and the above relationships were all significant at the 1% level. These results were consistent with Hypothesis 1, which indicated that an enterprise’s upstream and downstream innovations were conducive to improving the diversity and technical standards of an enterprise’s exported products, breaking the monopoly of developed countries and reducing the price of intermediate products. The competitors’ innovation also was conducive to improving the overall reputation and image of product manufacturing in the region and industry, thereby enhancing the exports of private enterprises.

In addition, the regression coefficients of control variables indicated that export propensity, volume and intensity had significant positive correlations with the age and size of enterprises, indicating that larger and more mature enterprises were more likely to export. The regression coefficients of financing constraints and capital intensity were significantly negative, indicating that financial constraints may hinder the exports of private enterprises.

### Robustness Test and Heterogeneity Analysis

#### Robustness Test

Based on the basic estimation model in Table 1, two robustness tests were conducted, and the results were given in Table 2. First, the intermediate input of the upstream industry to the industry and the intermediate output provided by the industry to the downstream industry were processed by the extrapolation and interpolation methods (Casciaro and Piskorski, 2005; Li et al., 2018), which linearly extrapolated the input-output data of other years by the input-output tables of 2002, 2007, and 2012 (Table 2, Model 1). Second, we expanded the sample range of private enterprises, to include all enterprises except foreign-funded enterprises and state-owned enterprises (Table 2, Model 2). Specifically, if the proportion of state-owned capital were more than 50%, it was regarded as a state-owned enterprise, and if the foreign capital were more than 25%, it was regarded as a foreign enterprise. The results in Table 2 report that, after expanding the sample range and changing the calculation method, the regression coefficients of upstream, downstream and degree of horizontal innovation on private enterprises’ export propensity, volume and intensity were all positive and significant at the 1% level. This indicated that the research findings in our paper are robust.

**TABLE 2:** Robustness tests.

Variables	Export propensity		Export volume		Export intensity	
Model	Robustness 1	Robustness 2	Robustness 1	Robustness 2	Robustness 1	Robustness 2
Upstream innovativeness	0.3190*** (0.008)	0.6124*** (0.008)	0.1468*** (0.004)	0.3849*** (0.005)	0.0018*** (0.000)	0.0054*** (0.000)
Control variables	Yes	Yes	Yes	Yes	Yes	Yes
Enterprise fixed effect	Yes	Yes	Yes	Yes	Yes	Yes
Year fixed effect	Yes	Yes	Yes	Yes	Yes	Yes
*R* ^2^	—	—	0.8467	0.8514	0.8691	0.8711
Downstream innovativeness	0.3082*** (0.008)	0.6173*** (0.008)	0.1397*** (0.004)	0.3962*** (0.005)	0.0015*** (0.000)	0.0048*** (0.000)
Control variables	Yes	Yes	Yes	Yes	Yes	Yes
Enterprise fixed effect	Yes	Yes	Yes	Yes	Yes	Yes
Year fixed effect	Yes	Yes	Yes	Yes	Yes	Yes
*R* ^2^	—	—	0.8466	0.8513	0.8691	0.8710
Horizontal innovativeness	0.4136*** (0.009)	0.3387*** (0.005)	0.0999*** (0.002)	0.0954*** (0.002)	0.0012*** (0.000)	0.0013*** (0.000)
Control variables	Yes	Yes	Yes	Yes	Yes	Yes
Enterprise fixed effect	Yes	Yes	Yes	Yes	Yes	Yes
Year fixed effect	Yes	Yes	Yes	Yes	Yes	Yes
*R* ^2^	—	—	0.8465	0.8502	0.8691	0.8710
*N*	102994	269529	762602	1568031	762039	1566322

Robust standard errors are reported in parentheses. ***p < 0.01. Logit models are used in (1) – (2).

It appears that after the expansion of the sample range of private enterprises in Table 2, the degree of impact of the regression coefficients on the exports of private enterprises was generally smaller in Table 2 than in Table 1. Thus, we speculate that the influence of stakeholders’ external innovation on the exports of private enterprises was larger than that of other types of enterprises, as the private enterprises in Table 2 may have included other types of capital such as state-owned and collective capital, which may be affected by factors such as government intervention. This may have inhibited the influence of the stakeholders’ external innovation on their exports. In contrast, purely private enterprises were more sensitive and responsive to market changes in business decision-making, so they could better adapt to the market’s role in allocating resources as well as in learning and obtaining knowledge and technical resources from the stakeholders’ external innovation. In other words, they seemed to have a stronger ability and motivation to improve their exports through learning and responding to the stakeholders’ external innovation.

#### Heterogeneity Analysis

We further analyzed the industrial and regional heterogeneities of the relationship between private enterprise exports and external innovation. Table 3 divides the samples into labor-intensive, capital-intensive and technological capital-intensive industries. The results showed that the external innovations of stakeholders (upstream innovativeness, downstream innovativeness and horizontal innovativeness) were significantly and positively correlated with private enterprises’ export propensity, volume and intensity, which indicates that the findings in our study are robust in different factor-intensive industries.

**TABLE 3:** Heterogeneity analysis by industry.

Models	(1)	(2)	(3)	(4)	(5)	(6)	(7)	(8)	(9)
			
Variables	Export propensity			Export volume			Export intensity		
Type	Labor intensive	Capital intensive	Capital and technology intensive	Labor intensive	Capital intensive	Capital and technology intensive	Labor intensive	Capital intensive	Capital and technology intensive
Upstream innovativeness	0.6926*** (0.022)	0.8721*** (0.022)	0.7743*** (0.028)	0.5231*** (0.014)	0.5980*** (0.012)	0.4326*** (0.016)	0.0058*** (0.001)	0.0069*** (0.000)	0.0054*** (0.001)
Control variables	Yes	Yes	Yes	Yes	Yes	Yes	Yes	Yes	Yes
Enterprise fixed effect	Yes	Yes	Yes	Yes	Yes	Yes	Yes	Yes	Yes
Year fixed effect	Yes	Yes	Yes	Yes	Yes	Yes	Yes	Yes	Yes
*R* ^2^	—	—	—	0.8342	0.8365	0.8372	0.8537	0.8649	0.8657
Downstream innovativeness	0.5438*** (0.019)	0.8728*** (0.022)	0.6556*** (0.026)	0.3706*** (0.012)	0.5905*** (0.012)	0.3461*** (0.014)	0.0032*** (0.001)	0.0065*** (0.000)	0.0042*** (0.001)
Control variables	Yes	Yes	Yes	Yes	Yes	Yes	Yes	Yes	Yes
Enterprise fixed effect	Yes	Yes	Yes	Yes	Yes	Yes	Yes	Yes	Yes
Year fixed effect	Yes	Yes	Yes	Yes	Yes	Yes	Yes	Yes	Yes
*R* ^2^	—	—	—	0.8334	0.8363	0.8368	0.8536	0.8649	0.8657
Horizontal innovativeness	0.3339*** (0.015)	0.5052*** (0.016)	0.5276*** (0.024)	0.1133*** (0.005)	0.0888*** (0.003)	0.1827*** (0.010)	0.0012*** (0.000)	0.0008*** (0.000)	0.0030*** (0.000)
Control variables	Yes	Yes	Yes	Yes	Yes	Yes	Yes	Yes	Yes
Enterprise fixed effect	Yes	Yes	Yes	Yes	Yes	Yes	Yes	Yes	Yes
Year fixed effect	Yes	Yes	Yes	Yes	Yes	Yes	Yes	Yes	Yes
*R* ^2^	—	—	—	0.8322	0.8324	0.8362	0.8536	0.8647	0.8657

Robust standard errors are reported in parentheses. ***p < 0.01. Logit models are used in (1) – (3).

Table 4 also classifies the regions where the sample enterprises were located into the eastern, central and western regions, and explores the effects of the external innovations of upstream and downstream industries and competitors in different regions on the exports of private enterprises. The results show that in the eastern and central regions, export propensity, volume and intensity of private enterprises were positively correlated with external innovations of upstream and downstream market stakeholders and competitors. However, the positive relationship between private enterprises’ export and external innovations in the western region was relatively weak: the relationships between private enterprises’ export propensity, volume, intensity and horizontal innovation in the western region were not significant, nor were the relationships between export intensity and upstream, downstream innovation.

**TABLE 4:** Heterogeneity analysis by region.

Models	(1)	(2)	(3)	(4)	(5)	(6)	(7)	(8)	(9)
			
Variables	Export propensity			Export volume			Export intensity		
Type	eastern region	Central region	Western region	eastern region	Central region	Western region	Eastern region	Central region	Western region
Upstream innovativeness	0.1517*** (0.018)	1.2836*** (0.034)	0.1881* (0.108)	0.0913*** (0.008)	1.0196*** (0.012)	0.0376** (0.017)	0.0040*** (0.000)	0.0090*** (0.000)	0.0001 (0.001)
Control variables	Yes	Yes	Yes	Yes	Yes	Yes	Yes	Yes	Yes
Enterprise fixed effect	Yes	Yes	Yes	Yes	Yes	Yes	Yes	Yes	Yes
Year fixed effect	Yes	Yes	Yes	Yes	Yes	Yes	Yes	Yes	Yes
*R* ^2^	—	—	—	0.8428	0.7592	0.7990	0.8589	0.8176	0.8183
Downstream innovativeness	0.0580*** (0.017)	1.4238*** (0.037)	0.3065*** (0.116)	0.0484*** (0.007)	1.0640*** (0.013)	0.0501*** (0.017)	0.0021*** (0.000)	0.0098*** (0.000)	0.0010 (0.001)
Control variables	Yes	Yes	Yes	Yes	Yes	Yes	Yes	Yes	Yes
Enterprise fixed effect	Yes	Yes	Yes	Yes	Yes	Yes	Yes	Yes	Yes
Year fixed effect	Yes	Yes	Yes	Yes	Yes	Yes	Yes	Yes	Yes
*R* ^2^	—	—	—	0.8427	0.7598	0.7990	0.8589	0.8177	0.8184
Horizontal innovativeness	0.0655*** (0.012)	0.6673*** (0.024)	–0.0029 (0.037)	0.0267*** (0.003)	0.1624*** (0.004)	0.0008 (0.004)	0.0010*** (0.000)	0.0014*** (0.000)	–0.0001 (0.000)
Control variables	Yes	Yes	Yes	Yes	Yes	Yes	Yes	Yes	Yes
Enterprise fixed effect	Yes	Yes	Yes	Yes	Yes	Yes	Yes	Yes	Yes
Year fixed effect	Yes	Yes	Yes	Yes	Yes	Yes	Yes	Yes	Yes
*R* ^2^	—	—	—	0.8427	0.7215	0.7980	0.8589	0.8162	0.8183

Robust standard errors are reported in parentheses. ***p < 0.01; **p < 0.05; *p < 0.1. Logit models are used in (1) – (3).

### Mechanism of Enterprise Innovation

In Hypothesis 2, we asserted that the relationships between the exports of private enterprises and external innovations of stakeholders would be affected by the mediating role of enterprise innovation, because enterprise innovation is an important way to improve the competitiveness of exported products and services (Cockburn et al., 2016). Moreover, external innovations of stakeholders promote enterprise innovation through mechanisms such as resource exchange, knowledge spillover and pressure transmission (Li et al., 2018). Accordingly, Table 5 examines the mediating effects of enterprise innovation in the relationship between three types of external innovation (upstream, downstream and horizontal) and the exports of private enterprises. The basic estimation results in Table 1 indicate that the main effects of these three types of external innovation on the exports of private enterprises were significantly and positively correlated at the 1% level.

**TABLE 5:** The mediating effect tests of enterprise innovation.

Models	(1)	(2)	(3)	(4)	(5)	(6)	(7)	(8)	(9)	(10)
				
Variables	Innovation	Export propensity			Export volume			Export intensity		
Upstream Innovativeness	0.3572 *** (0.005)	0.4542*** (0.014)			0.3909*** (0.006)			0.0044*** (0.000)		
Downstream innovativeness	0.2797*** (0.000)		0.3993*** (0.013)			0.3500*** (0.006)			0.0036*** (0.000)	
Horizontal innovativeness	0.1048*** (0.000)			0.2081*** (0.009)			0.0802*** (0.002)			0.0007*** (0.000)
Enterprise innovation		0.2754** (0.005)	0.2861*** (0.005)	0.3040*** (0.005)	0.2065** (0.003)	0.2117*** (0.003)	0.2207*** (0.003)	0.0041*** (0.000)	0.0042*** (0.000)	0.0043*** (0.000)
Control variables	Yes	Yes	Yes	Yes	Yes	Yes	Yes	Yes	Yes	Yes
Enterprise fixed effect	Yes	Yes	Yes	Yes	Yes	Yes	Yes	Yes	Yes	Yes
Year fixed effect	Yes	Yes	Yes	Yes	Yes	Yes	Yes	Yes	Yes	Yes
Mediating effect		—	—	—	15.87%	14.47%	22.39%	24.82%	24.99%	37.55%
*N*	701171	102319	102319	102319	701171	701171	701171	700677	700677	700677
*R* ^2^					0.8370	0.8367	0.8355	0.8591	0.8590	0.8590

Robust standard errors are reported in parentheses. ***p < 0.01; **p < 0.05; *p < 0.1. Logit models are used in (2) – (4).

Based on the above findings, Model (1) in Table 5 shows that the regression coefficients of enterprise innovation and the three types of external innovation were significantly positively correlated at the 1% level. And Models (2) – (4) indicate that enterprise innovation had positive effects on private enterprises’ export propensity, which indicated that enterprise innovation played a mediating role in the relationships between the three types of external innovations and the export propensity of the private enterprises. As the regression coefficients of the three types of external innovations also were significant, enterprise innovation played a partially mediating role.

Similarly, in Table 5, Models (5)–(10) indicate that the partial mediating effects of enterprise innovation were also supported in the relationships between the three types of external innovations of the stakeholders and the export volume and export intensity of private enterprises, and this effect ranged from 14.47 to 37.55%. These results indicate that the external innovations of market stakeholders not only directly promoted the exports of private enterprises, but also indirectly promoted the exports of private enterprises by improving enterprise innovation.

## Conclusion and Implications

Based on the theoretical perspective of market stakeholders, this paper explored the effects of external innovations on the exports of private enterprises. We found that the external innovations of upstream suppliers, downstream customers and competitors significantly promoted the export propensity, volume and intensity of private enterprises. The heterogeneity analysis further illustrated that our results were robust in different factor-intensive industries. Moreover, compared with the western region, the positive effects of external innovations in the eastern and central regions on the exports of private enterprises were even more significant. In addition, we found that enterprise innovation was an important mediator in the relationships between stakeholders’ external innovations and the exports of private enterprises.

Our research findings have several important policy implications. First, we found that the external innovation of stakeholders played important roles in stimulating the exports of private enterprises. This suggested that with the intensification of global trade disputes and the rapid rise of labor and land factor costs, it would be difficult to continue expanding exports by relying on low value-added activities. Thus, our findings highlight the necessity and urgency of maintaining and enhancing export competitiveness by promoting innovation. Therefore, government should firmly implement the innovation-driven strategy. More importantly, the relevant industrial policy incentives released by government should focus on a small number of key enterprises and aim to improve the innovation technology and technological level of the overall industrial chain. In particular, policies should focus on high-tech small and medium-sized enterprises in the upstream and downstream industries of key exported products and their product activities expected to make breakthroughs in basic components, special materials and key processes. By solving the weak links in the upstream and downstream industrial chains, we assert that an enterprise can achieve a higher position in the global value chain and thus enjoy more sustainable exports.

Second, we found that the positive effects of external innovations on the exports of private enterprises in the western region were weak, indicating that the channels for private enterprises in the western region to obtain new technologies, knowledge and talents from their stakeholders’ external innovations were relatively blocked. This negatively impacted their export competitiveness. One important reason for these findings is that local governments in the western region were more likely to intervene in enterprise activities, resulting in a distortion of factor allocation and limiting the ability and motivation of private enterprises to obtain and transform innovative resources. Therefore, when implementing export and innovation industrial policies in the western region, it is necessary to coordinate the forces of the government and the market, so that the resources needed for innovation – such as new technologies, processes, information and talents – can be exchanged more efficiently at the industrial and supply chain levels. This can enhance the overseas competitiveness of exported products and services. In addition, private enterprises should maintain close relationships with the upstream and downstream enterprises as well as pay attention to and track the innovation progress of the upstream and downstream enterprises and competitors. Overall, they can transform the external innovation achievements of stakeholders into a major force to improve the technological complexity and price competitiveness of their exports.

We used data from the 1998 to 2010 China Industrial Enterprise Database in this study. However, with the change of trade environment and economic development level, the relationship between external innovation and exports of private enterprises may be influenced. Therefore, whether our findings can explain the latest corporate practices requires further support from updated data.

## Data Availability Statement

The data analyzed in this study is subject to the following licenses/restrictions: Data analyzed in this study is from China industrial enterprise database released by the national bureau of statistics, which can only be obtained by purchasing database. Requests to access these datasets should be directed to JH, haojie@mail.zjgsu.edu.cn.

## Author Contributions

SC and JH designed the research model. SC, XJ, and YW wrote the manuscript. XJ analyzed data. All authors approved the manuscript for publication.

## Conflict of Interest

The authors declare that the research was conducted in the absence of any commercial or financial relationships that could be construed as a potential conflict of interest.

## Publisher’s Note

All claims expressed in this article are solely those of the authors and do not necessarily represent those of their affiliated organizations, or those of the publisher, the editors and the reviewers. Any product that may be evaluated in this article, or claim that may be made by its manufacturer, is not guaranteed or endorsed by the publisher.
